# Data on the positive synergic action of dimethylacetamide and trehalose on quality of cryopreserved chicken sperm

**DOI:** 10.1016/j.dib.2016.11.059

**Published:** 2016-11-22

**Authors:** Fabio Mosca, Manuela Madeddu, Ahmad Abdel Sayed, Luisa Zaniboni, Nicolaia Iaffaldano, Silvia Cerolini

**Affiliations:** a Department of Veterinary Medicine, University of Milan, via Trentacoste 2, 20134 Milan, Italy; b Department of Agricultural, Environmental and Food Science, University of Molise, via De Sanctis, 86100 Campobasso, Italy

## Abstract

This data article contains supporting information regarding the research article entitled “Combined effect of permeant and non-permeant cryoprotectants on the quality of frozen/thawed chicken sperm”(Mosca et. al., 2016) [1]. The combined effect of the permeant cryoprotectants agent dimethylacetamide and the non-permeant cryoprotectants agent trehalose on the quality of frozen-thawed chicken semen was assessed. In particular, the quantitative dimethylacetamide/trehalose ratio was investigated freezing semen samples according to the following treatments: trehalose 0.1 M+0% dimethylacetamide (DMA-0), trehalose 0.1 M+3% dimethylacetamide (DMA-3), trehalose 0.1 M+6% dimethylacetamide (DMA-6).

**Specifications Table**

**Table:** 

Subject area	*Biology, Animal Science*
More specific subject area	*Cryoconservation of chicken semen*
Type of data	*Table*
How data was acquired	*Fluorescence microscopy, SCA (Sperm Class Analyzer)*
Data format	*Analyzed*
Experimental factors	*The natural osmoprotectant trehalose (0.1 M) was combined with different level (0–6%) of the permeant cryoprotectant dimethylacetamide to prevent cryodamages in chicken semen.*
Experimental features	*Sperm quality was assessed before and after freezing/thawing in chicken semen processed for cryopreservation using a range of quantitative dimethylacetamide/trehalose ratios to identify the most effective cryoprotective combination.*
Data source location	*Milano, Lodi (Italy)*
Data accessibility	*Data is available with this article*

**Value of the data**

• Data presented in this paper confirm a positive synergic action of dimethylacetamide and trehalose on quality of frozen-thawed chicken sperm.
• These data encourage the investigation on the interaction between permeating cryoprotectants, like dimethylacetamide, and natural osmoprotectants, such as trehalose, to improve the success of sperm cryopreservation in birds.
• These data contribute for designing further experiments aiming to identify a chicken semen cryopreservation reference procedure.

## 1 Data

Data include all sperm quality parameters recorded in fresh and cryopreserved chicken semen (Table 1) and the recovery rates of viable and motile sperm after freezing–thawing (Table 2). The most effective cryoprotectant combination includes both trehalose and DMA; in contrast, the absence of DMA (DMA-0) is responsible for more severe loss in sperm quality.

## 2 Experimental design, materials and methods

### 2.1 Bird management and semen collection

Twenty-seven adult Lohmann male fowl (*Gallus gallus domesticus*) were housed at 28 weeks of age in individual cages and handled in accordance with the principles presented in Guidelines for the Care and Use of Agricultural Animals in Research and Teaching [2]. Semen was collected according to the technique initially described by Burrows and Quinn [3]. Each day of collection, males were divided in three different groups (nine birds/group) and all ejaculates collected within one group were pooled into one semen sample.

### 2.2 Semen processing for cryopreservation

Each pooled semen sample was split into three aliquots, each one assigned to one treatment. Semen aliquots were diluted to a concentration of 1.5×10^9^ sperm/ml using Lake pre-freezing modified extender (8 g D-fructose, 5 g potassiumacetate,19.2 g sodium glutamate, 3 g polyvinylpyrrolidone, 0.7 g magnesium acetate, 3.75 g glycine, adjusted to 1 L with distilled water; pH 7.0, osmolality 340 mOsmol/kg) added with 0.1 M trehalose. The diluted semen was immediately cooled and kept at 4 °C for 30 min. During this incubation, semen samples were transferred to the laboratory for further quality assessment and freezing processing. Sperm quality assessment included viability and motility. Sperm viability was measured using the dual fluorescent staining SYBR14/propidium iodide (PI) procedure (LIVE/DEAD Sperm Viability Kit, Molecular Probes, Invitrogen), as described by Rosato and Iaffaldano [4] with minor modifications. Sperm motility was assayed using a computer-aided sperm analysis system employing the Sperm Class Analyzer (SCA) software (version 4.0, Microptic S.L., Barcelona, Spain). After the assessment of sperm quality, semen aliquots were further diluted to 1×10^9^ sperm/ml with the extender containing dimethylacetamide (DMA) to three different final DMA concentration: 6% dimethylacetamide (DMA-6), 3% (DMA-3) and 0% (DMA-0). Semen was finally loaded into 0.25 ml French straws (three different straw colours were used according to the three different treatments). Straws were transferred on racks floating over a nitrogen bath at 3 cm of height [5] and frozen for 10 min. Straws were stored in cryotank for at least 7 days. Semen collection was repeated on four days to process 12 pooled semen samples (12 replicates per treatment) and a total of 24 straws were stored per treatment. The straws were thawed in water bath at 38 °C for 30 s and sperm quality was assessed in thawed semen.

### 2.3 Statistical analysis

Analysis of variance on sperm quality parameters recorded in fresh and frozen/thawed semen samples was performed using the MIXED procedure of SAS [6]. Treatment (DMA-6, DMA-3, DMA-0), time (fresh semen; thawed semen), and the relative interaction (treatment * time) were considered as fixed effects and the pooled semen sample was considered as random effect. The *t*-test was used to compare LSMeans.

The recovery rates (%) of sperm viability after cryopreservation were calculated as follows: [(mean on thawed semen*100)/mean on fresh semen]. The same formula was used to calculate the recovery rates (%) of sperm motility and progressive motility after cryopreservation. Analysis of variance on the recovery variables was performed using the GLM procedure of SAS [6], and the treatment was the only source of variation included in the model. The *t*-test was used to compare LSMeans.

## Figures and Tables

**Table 1:** Sperm quality parameters (LSMeans±SE) measured in fresh semen and in semen frozen according the following treatments: 0.1 M trehalose+0% dimethylacetamide (DMA-0), 0.1 M trehalose+3% dimethylacetamide (DMA-3), 0.1 M trehalose+6% dimethylacetamide (DMA-6).

**Sperm parameters**^a^	**Fresh**	**DMA-0**	**DMA-3**	**DMA-6**	**S.E.**
Viability (%)	87.9^A^	4.3^B^	31.8^C^	37.1^C^	2.0
Motility (%)	81.7^A^	8.0^B^	24.2^C^	29.1^C^	2.1
Progressive motility (%)	14.1^A^	0.1^B^	1.5 ^B^	1.2 ^B^	1.3
VCL (µm/s)	47.4^A^	25.7^B^	35.6^C^	33.7^C^	1.5
VSL (µm/s)	17.0^A^	4.6^B^	10.1^C^	9.3^C^	0.8
VAP (µm/s)	28.3^A^	10.2^B^	18.4^C^	17.8^C^	1.0
LIN (%)	35.7^A^	17.9^B^	28.1^C^	27.7^C^	1.0
STR (%)	59.8^A^	45.2^B^	54.4^C^	52.5^C^	1.1
WOB (%)	59.6^A^	39.3^B^	51.6^C^	52.7^C^	0.9
ALH (µm)	2.8^A^	0.9^B^	2.5^C^	2.7^A^	0.1
BCF (Hz)	7.9^A^	0.7^B^	6.1^C^	5.4^C^	0.4

^A,B^ Values within each row with different superscript letters are significantly different (*p*<0.001).

a Viability, the percentage of viable spermatozoa; motility, the percentage of motile spermatozoa; progressive motility, spermatozoa swim forward fast in a straight line; VCL, curvilinear velocity; VSL, straight-line velocity; VAP, average path velocity; LIN (VSL/VCL×100), linearity; STR (VSL/VAP×100) straightness; WOB (VAP/VCL×100); ALH, amplitude of lateral head displacement; BCF, beat cross frequency.

**Table 2:** Recovery rates (LSMeans±SE) of sperm quality recorded in semen frozen with three different treatments: 0.1 M trehalose+0% dimethylacetamide (DMA-0), 0.1 M trehalose+3% dimethylacetamide (DMA-3), 0.1 M trehalose+6% dimethylacetamide (DMA-6).

	**DMA-0**	**DMA-3**	**DMA-6**	**S.E.**
Viability (%)	4.9^A^	36.5^B^	42.4^B^	2.8
Motility (%)	9.8^A^	30.2^B^	36.0^B^	2.5
Progressive motility (%)	1.3^A^	19.0^A^	15.6^A^	5.6

^A,B^ Values within a row with different superscripts differ significantly at *p*<0.001.
